# Besuche von minderjährigen Angehörigen in der Intensiv- und Notfallmedizin: Kinder als Besucher willkommen!

**DOI:** 10.1007/s00063-023-01004-z

**Published:** 2023-04-19

**Authors:** Maria Brauchle, Teresa Deffner, Alexander Brinkmann, Svenja Dehner, Rolf Dubb, Simon Finkeldei, Birga Gatzweiler, Carsten Hermes, Christian Heyd, Magdalena Hoffmann, Marie-Madlen Jeitziner, Arnold Kaltwasser, Tita Kern, Kathrin Knochel, Lars Krüger, Heiner Melching, Guido Michels, Tilmann Müller-Wolff, Sabrina Pelz, Julian Rudolph, Denise Schindele, Anna-Henrikje Seidlein, Arne Simon, Marina Ufelmann, Peter Nydahl

**Affiliations:** 1 Klinik für Anästhesie und Intensivmedizin, Landeskrankenhaus Feldkirch, Feldkirch, Österreich; 2 Klinik für Anästhesie und Intensivmedizin, Universitätsklinikum Jena, Jena, Deutschland; 3 Klinik für Anästhesie, operative Intensivmedizin und spezielle Schmerztherapie, Kliniken Landkreis Heidenheim gGmbH, Heidenheim, Deutschland; 4 Klinik fürAnästhesiologie, Intensiv- und Schmerztherapie mit Abteilung Palliativmedizin, Klinikum St. Georg gGmbH, Leipzig, Deutschland; 5 Akademie der Kreiskliniken Reutlingen GmbH, Reutlingen, Deutschland; 6 KinderKrisenIntervention, der AETAS Kinderstiftung, München, Deutschland; 7 Ludwig-Maximilians-Universität Klinikum Kinderpalliativzentrum München, München, Deutschland; 8 Bonn, Deutschland; 9 Weiterbildung in den Gesundheitsfachberufen, Universitätsmedizin Mainz, Mainz, Deutschland; 10 Klinische Abteilung für Endokrinologie und Diabetologie, Universitätsklinik für Innere Medizin/Research Unit for Safety in Health, Klinische Abteilung für Plastische, Ästhetische und Rekonstruktive Chirurgie, Universitätsklinik für Chirurgie, Medizinische Universität Graz, Graz, Österreich; 11 Stabsstelle Qualitätsmanagement-Risikomanagement, Landeskrankenhaus(LKH)-Universitätsklinikum Graz, Graz, Österreich; 12 Universitätsklinik für Intensivmedizin, Universitätsspital Bern (Inselspital), Universität Bern, Bern, Schweiz; 13 Akademie der Kreiskliniken Reutlingen GmbH, Reutlingen, Deutschland; 14 KinderKrisenIntervention der AETAS Kinderstiftung, München, Deutschland; 15 Klinikum rechts der Isar, Technische Universität München, München, Deutschland; 16 Herz- und Diabeteszentrum NRW, Universitätsklinikum der Ruhr-Universität Bochum, Bad Oeynhausen, Deutschland; 17 Deutsche Gesellschaft für Palliativmedizin, Berlin, Deutschland; 18 Klinik für Akut- und Notfallmedizin, St.-Antonius-Hospital gGmbH, Eschweiler, Deutschland; 19 Hochschule für angewandte Wissenschaften München, München, Deutschland; 20 Universitäts- und Rehabilitationskliniken Ulm, Ulm, Deutschland; 21 Abteilung für Anästhesie und Intensivmedizin, Landeskrankenhaus Feldkirch, Feldkirch, Österreich; 22 Regionale Kliniken Holding, Klinikum Ludwigsburg, Ludwigsburg, Deutschland; 23 Institut für Ethik und Geschichte der Medizin, Zentrum für Intensiv- und Überwachungspflege, Universitätsmedizin Greifswald, Greifswald, Deutschland; 24 Klinik für Pädiatrische Onkologie und Hämatologie, Universitätsklinikum des Saarlandes, Homburg/Saar, Deutschland; 25 Pflegedirektion/Bildungszentrum Klinikum rechts der Isar, Technische Universität München, München, Deutschland; 26 Pflegeforschung und -entwicklung, Universitätsklinikum Schleswig-Holstein Kiel, Kiel, Deutschland

**Keywords:** Familienunterstützung, Heranwachsende, Intensivstationen, Notaufnahmen, Psychosoziales Unterstützungssystem, Family support, Adolescents, Intensive care units, Emergency departments, Psychosocial support system

## Abstract

In diesem Empfehlungspapier werden zentrale Vorschläge für den Besuch von Kindern auf Intensivstationen (Pädiatrie und Erwachsenenbereich), Intermediate-care-Stationen und in Notaufnahmen vorgestellt. Auf Intensivstationen und in Notaufnahmen im deutschsprachigen Raum werden die Besuchsregelungen für Kinder und Jugendliche sehr heterogen gestaltet. Mitunter dürfen sie ohne Begrenzungen in Alter und Dauer Patient:innen besuchen, manchmal ist dies erst ab dem Teenageralter und nur für kurze Dauer möglich. Ein Besuchswunsch von Kindern löst beim Personal oftmals unterschiedliche, teilweise ablehnende Reaktionen aus. Leitungen sind aufgefordert, diese Haltung gemeinsam mit ihren Mitarbeiter:innen zu reflektieren und eine Kultur der familienorientierten Versorgung zu entwickeln. Obwohl die Evidenz für Vorteile durch Kinder als Besuchende begrenzt ist, spricht mehr für als gegen einen Besuch, auch in hygienischer, psychosozialer, ethischer, religiöser und kultureller Hinsicht. Dennoch ist keine pauschale Empfehlung für oder gegen einen Besuch möglich. Die Entscheidungen für Besuche sind komplex und bedürfen sorgfältiger Überlegungen und Abwägungen.

## Abstract (en)

In this white paper, key recommendations for visitation by children in intensive care units (ICU; both pediatric and adult), intermediate care units and emergency departments (ED) are presented. In ICUs and EDs in German-speaking countries, the visiting policies for children and adolescents are regulated very heterogeneously: sometimes they are allowed to visit patients without restrictions in age and time duration, sometimes this is only possible from the age of teenager on, and only for a short duration. A request from children to visit often triggers different, sometimes restrictive reactions among the staff. Management is encouraged to reflect on this attitude together with their employees and to develop a culture of family-centered care. Despite limited evidence, there are more advantages for than against a visit, also in hygienic, psychosocial, ethical, religious, and cultural aspects. No general recommendation can be made for or against visits. The decisions for a visit are complex and require careful consideration.

## Hintergrund

### Infobox Begriffsdefinitionen

- *Kinder* sind alle Personen bis einschließlich zum 18. Lebensjahr Früh- und Neugeborene, Kleinkinder, Schulkinder und Jugendliche.
- *Eltern *schließt sonstige rechtlich-verbindliche Bezugspersonen und Sorgeberechtigte mit ein.
- *Angehörige* sind neben Lebenspartner:innen und Verwandten – zu denen auch minderjährige Kinder gehören – auch relevante Freund:innen, Kolleg:innen, Nachbar:innen oder andere Personen. Angehörige sind alle Personen, zu denen Patient:innen eine bedeutsame Beziehung haben.
- *Psychosoziale Fachkräfte* sind in dieser Empfehlung v. a. Fachpersonen mit Expertise in psychotraumatologischen, entwicklungs- und palliativpsychologischen Aspekten sowie der Kommunikationsgestaltung mit Kindern.
- *Intensivstationen *sind Intensivstationen für Erwachsene, pädiatrische Intensivstationen, Intermediate-care(IMC)-Stationen und Notaufnahmen.

Seit vielen Jahren wird diskutiert, ob Kinder ihre erkrankten Eltern, Großeltern, Freund:innen und andere Angehörige (für den Text relevante Begriffsdefinitionen s. Infobox 1) auf der Intensivstation besuchen dürfen^1^. Was auf pädiatrischen Intensivstationen vielerorts Alltag ist – der Besuch von Geschwisterkindern – ist im Erwachsenenbereich Gegenstand andauernder Kontroverse. Der Zugang von Kindern auf Intensivstationen war von jeher eingeschränkt, was auf der Annahme basierte, dass Kinder mit der Gesamtsituation auf der Intensivstation möglicherweise nicht zurechtkommen und durch die Eindrücke traumatisiert werden könnten [7, 18]. Hinzu kommt die Vermutung, dass durch den Besuch von Kindern die Arbeit des Intensivpersonals beeinträchtigt wird, da Zeit und Energie aufgewendet werden müssen, die sonst in die Betreuung der Patient:innen fließen würden [4]. Zudem könnten die Patient:innen gestresst und sogar gefährdet werden, z. B. durch ein erhöhtes Infektionsrisiko [17]. Es liegen sowohl Argumente für als auch gegen den Besuch von Kindern vor. Ein Besuch kann schaden, ein Nichtbesuch kann ebenfalls schaden. In den meisten Fällen gibt es Argumente, die zeigen: Kinder können Besuche sehr wohl verarbeiten, wenn sie altersgerecht informiert werden. Kinder stellen auch kein erhöhtes Infektionsrisiko dar, wenn die geltenden Hygieneanforderungen eingehalten werden. Es gibt aber auch Ausnahmen, in denen Kinder die Situation trotz vorbereitender Gespräche nicht bewältigen konnten und den Besuch als traumatisierend erlebten. Doch dürfen diese Ausnahmen als Begründung verwendet werden, um Kindern generell den Besuch zu verwehren?

Altersgerecht informierte Kinder können Besuche auf Intensivstationen verarbeiten

Die Besuchsregelungen auf den Intensivstationen im deutschsprachigen Raum sind sehr heterogen und werden durch die Haltung des Personals bestimmt – und das nicht erst seit der Pandemie durch Coronavirus Disease 2019 (COVID-19). Leitungen sind aufgefordert, diese Haltung gemeinsam mit ihren Mitarbeiter:innen zu reflektieren und eine Kultur der familienorientierten Versorgung zu entwickeln und zu implementieren. Hierbei wird für die Etablierung von Kinderbesuchen auf Intensivstationen eine zielgerichtete und konsequente Unterstützung durch die Krankenhausleitung benötigt. Die nun vorliegenden Empfehlungen „Kinder als Angehörige und Besuchende“ haben zum Ziel, Mitarbeiter:innen aller Professionen, aber auch Eltern und Sorgeberechtigten eine Hilfestellung an die Hand zu geben, um einheitliche Regelungen für Intensivstationen zu entwickeln und zu implementieren. Die Empfehlungen wurden in einem Konsensprozess entwickelt und beruhen auf der bestmöglichen Evidenz.

Die Empfehlungen wurden im Konsens entwickelt und beruhen auf der bestmöglichen Evidenz

Die Empfehlungen wurden (verblindet) von der Arbeitsgruppe „ICU Kids“ unter der Leitung von Maria Brauchle, Teresa Deffner und Peter Nydahl in Kooperation mit den Sektionen psychologische Versorgungsstrukturen, Pflegeforschung und Pflegequalität, pädiatrische Intensiv- und Notfallmedizin, Ethik, Bewusstseinsstörungen und Koma sowie Sepsis und Infektiologie der Deutschen Interdisziplinären Vereinigung für Intensiv- und Notfallmedizin (DIVI) entwickelt [5, 6]. Darüber hinaus haben sich folgende Fachgesellschaften daran beteiligt: AETAS Kinderstiftung, Deutsche Gesellschaft für Palliativmedizin, Deutschsprachige Gesellschaft für Psychotraumatologie, Deutsche Gesellschaft für Interdisziplinäre Notfall- und Akutmedizin. Dieses Expertengremium unterschiedlicher Professionen und Disziplinen hat es sich zur Aufgabe gemacht, einen wissenschaftlich und ethisch begründeten Rahmen für eine einheitliche Besuchsregelung von Kindern auf Intensivstationen zu gestalten.

Die Autor:innen hoffen, dass diese Empfehlungen Verbreitung finden und Anstoß sind, um die Praxis zum Besseren zu verändern.

## Hygienische Voraussetzungen

Die Sorge vor einem erhöhten Infektionsrisiko der Patient:innen war und ist ein häufiges Argument gegen den Besuch von Kindern auf der Intensivstation. Jedoch lässt sich festhalten: Unter hygienischen Aspekten ist ein Kinderbesuch unbedenklich, wenn die im Folgenden beschriebenen Hygieneanforderungen eingehalten werden. Dies wird auch in Studien bestätigt, die die Sicherheit und die gesundheitlichen Ergebnisse von nichtrestriktiven und restriktiven Besuchsregelungen untersuchten (u. a. [9]).

Besuchende Kinder müssen altersgerecht in den notwendigen Hygienemaßnahmen unterwiesen werden

Allerdings müssen besuchende Kinder in altersentsprechender Form in den notwendigen Hygienemaßnahmen unterwiesen werden. Die wichtigste Hygienemaßnahme stellt die Händehygiene dar. Kinder ab dem Schulalter sind in der Lage, die Händedesinfektion nach Anleitung korrekt selbstständig durchzuführen. Bei Kleinkindern kann die erwachsene Bezugsperson die Hände des Kinds zwischen den eigenen Händen desinfizieren. Schutzkittel sind in der Basishygiene nicht indiziert [11].

Beim Besuch von infizierten, immunsupprimierten Patient:innen oder auf speziellen infektiologischen Intensivstationen sind möglicherweise erweiterte Hygienemaßnahmen erforderlich. Dies kann bedeuten, dass Schutzkittel, Handschuhe, Haube, Schutzbrille und Mund-Nasen-Schutz notwendig werden. Ob ein Besuch des Kinds unter diesen Bedingungen möglich und sinnvoll ist, hängt von der individuellen Situation ab und auch davon, ob die Schutzkleidung der Größe des Kinds entspricht. Bei relevanten Krankheitszeichen aufseiten des besuchenden Kinds sollte grundsätzlich von einem Besuch abgeraten werden [19, 11, 12, 2]. Dies gilt allerdings auch für Besuchende jeglichen Alters und nicht ausschließlich für Kinder. In diesem Fall kann alternativ ein virtueller Besuch erwogen werden.

## Psychotraumatologie

Von einer kritischen Erkrankung ist das gesamte Familiensystem betroffen. Insbesondere die Erkrankung von wichtigen Bezugspersonen kann – unabhängig von einem Besuch – die körperliche und psychische Entwicklung von Kindern beeinträchtigen. Gleichzeitig verfügen Kinder und Familien über verschiedene Ressourcen und Resilienzfaktoren [1].

Der Besuch der erkrankten Bezugsperson auf der Intensivstation kann für ein Kind psychotraumatologisch eine wertvolle, korrigierende Erfahrung beim Erleben von Hilflosigkeit, Kontrollverlust und Entsetzen sein [10]. Ein Besuch kann aber auch zu zusätzlicher Belastung führen. Um über die Sinnhaftigkeit eines Besuchs zu entscheiden, sollten zum einen die Möglichkeiten zur Vor- und Nachbereitung geprüft und zum anderen auch immer die individuellen Ressourcen und Risikofaktoren des gesamten Familiensystems eingeschätzt werden.

Kinder können durchaus mit belastenden Situationen umgehen

Kinder können durchaus mit belastenden Situationen umgehen. Sie verarbeiten oftmals das Erlebte im Spiel und drücken ihre Gedanken in Bildern oder Gebasteltem aus, mitunter fragen sie auch direkt. Abhängig vom Entwicklungsstand des Kinds sollte nach eigenen Phantasien und Vorstellungen gefragt werden und auf diesem Niveau mit dem Kind gesprochen werden.

Die Verarbeitung hochbelastender Erfahrungen durch potenziell traumatisierende Ereignisse vollzieht sich im Zeitverlauf. Die Frage nach gesunder Verarbeitung oder Erkrankung steht im Zusammenhang mit prä-, peri- und posttraumatischen Faktoren. Zudem wird sie durch die kognitiv-emotionale Entwicklung des Kinds beeinflusst [16].

Eltern sollten Informationen zur kindlichen Verarbeitung nach belastenden Ereignissen erhalten

Es entwickelt sich aber nicht aus jeder potenziell traumatisierenden Erfahrung eine Störung [8]. Eltern sollten daher Informationen zur kindlichen Verarbeitung nach belastenden Ereignissen erhalten und ermutigt sowie gestärkt werden, mit ihrem Kind über die aktuelle Situation der schweren Erkrankung zu sprechen. Dies kann z. B. altersgemäß im Spiel aufgegriffen werden, um die kindliche Verarbeitung zu unterstützen, mögliche Zeichen psychischer Belastung zu erkennen und als Eltern auf die geäußerten Bedürfnisse des Kinds eingehen zu können.

## Juristische Perspektive

Das Besuchsrecht innerhalb eines Krankenhauses – und damit auch mögliche Altersbeschränkungen – werden meist über das Hausrecht reguliert. Jeweilige Regelungen zum Sorge- und Betreuungsrecht sind selbstverständlich zu beachten. Es gibt verschiedene rechtliche Konstellationen aus der Praxis, bei denen es möglich wäre, dass ein Kind eine eigene unabhängige Entscheidung treffen möchte oder muss. Auf den meisten Intensivstationen im deutschsprachigen Raum liegt das mediane Alter, ab dem Kinder als Besuchende akzeptiert werden, bei 12 Jahren; ab diesem Alter ist für die Jugendlichen ein Besuch meist möglich.

Bei jüngeren Kindern gilt in der Regel die Entscheidung der Eltern

Bei jüngeren Kindern gilt in der Regel die Entscheidung der Eltern. Damit gelten die vorliegenden Empfehlungen für die meisten Konstellationen. Es kann aber auch zu schwierigen innerfamiliären Verhältnissen kommen, bei denen individuell entschieden werden muss – z. B. wenn eine nichtgeschäftsfähige Patient:in gleichzeitig die sorgeberechtigte Person ist. Ist die notwendige geistige Reife beim Kind erkennbar, sollten dessen Wünsche bei der Besucherreglung mitberücksichtigt werden. Viele Entscheidungen hängen nicht expliziert vom kalendarischen Alter ab.

## Kulturwandel

Um einen Kulturwandel Richtung familienzentrierter Versorgung gewährleisten zu können, sollte das Leitungspersonal entsprechende Schulungen organisieren. Pflegende, ärztlicher Dienst und nichtmedizinisches Personal sowie alle Stakeholder:innen im direkten Umfeld der Intensivstation sollten zum Einbezug von minderjährigen Angehörigen in der Intensiv- und Notfallmedizin informiert und geschult werden. Die Teilnahme an Schulungen sollte auch den Mitarbeiter:innen ermöglicht werden, die sich in Ausbildung befinden. Denn gerade in der Ausbildung wie auch im Studium wird der Umgang mit Kindern als Angehörige oft lückenhaft behandelt [3, 18]. Es eignen sich hier für die Schulung aller Mitarbeitenden Workshops und Trainings, Kurzfortbildungen oder One Minute Wonder [13, 14].

Schulungsmaterialien für Familien und Kinder in Form von Flyern, Postern und Videos werden zurzeit von der Autorengruppe gesammelt bzw. erstellt.

## Empfehlungen auf einen Blick

**Figure 1:**
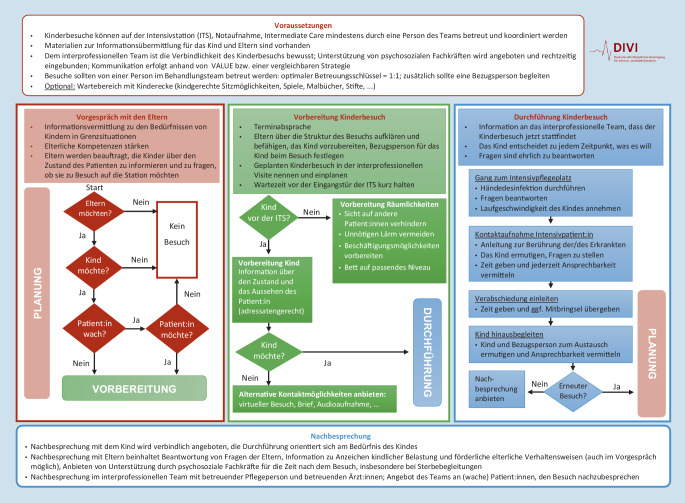

Im Folgenden werden die zentralen Empfehlungen zum Besuch von Kindern auf der Intensivstation in 10 Aussagen aufgeführt. Zudem sind in Abb. 1 die Voraussetzungen für einen Kinderbesuch und ein Algorithmus zur Planung und Durchführung des Aufenthalts in einem Schaubild dargestellt. Die erforderliche Kommunikation unter allen Beteiligten sollte durchgängig anhand des Akronyms VALUE [15] erfolgen:

- „Value“: empathische Gesprächsführung;
- „Acknowledge“: Akzeptanz des Gegenübers;
- „Listen“: geduldiges Zuhören;
- „Understand“: an der Person und ihrer Situation orientiertes Verstehen;
- „Elicit family questions“: zu eigenen Fragen motivieren.

### Empfehlung 1: den Besuch von Kindern im interprofessionellen Team planen

Der Besuch sollte schrittweise geplant und als Tagesziel individuell formuliert und dokumentiert werden. Das verbessert die Kommunikation im Team und beugt Missverständnissen vor. Eine notwendige Unterstützung von psychosozialen Berufsgruppen sollte rechtzeitig angefordert werden.

### Empfehlung 2: elterliche Kompetenzen stärken

Die Entscheidung über einen Besuch von Kindern auf der Intensivstation steht und fällt mit der Befähigung der Eltern. Sie sind diejenigen, die ihr Kind fragen, ob es zu Besuch auf die Intensivstation möchte, und die eng in den Kinderbesuch eingebunden werden. Um diese Aufgabe erfüllen zu können, sollten die Eltern in Gesprächen vorbereitet und in ihren Kompetenzen gestärkt werden.

### Empfehlung 3: kindgerechte Information sicherstellen

Das Kind sollte dem Besuch auf der Intensivstation zustimmen und altersgerecht vorbereitet und begleitet werden. Die Informationen sollten sich zum einen am kognitiven und emotionalen Entwicklungsalter sowie am Wissensdurst des Kinds orientieren, zum anderen an der aktuellen medizinischen Situation der Patientin bzw. des Patienten. Die Kommunikation sollte immer von Ehrlichkeit, Transparenz und Wertschätzung geprägt sein und Sicherheit, Orientierung, Verbindung sowie Kontrolle vermitteln.

### Empfehlung 4: den Besuch von Kindern vorbereiten, begleiten und nachbereiten

Der Besuch von Kindern wird von einer festgelegten Person im Behandlungsteam betreut. Der Termin sollte allen Beteiligten bekannt, das Patientenzimmer vorbereitet sein. Wichtig ist, für eine möglichst ruhige Umgebung zu sorgen. Die Wartezeit vor der Eingangstür sollte z. B. kurz gehalten, der Blick auf andere Patient:innen möglichst verhindert werden. Nach jedem Besuch des Kinds sollte im Behandlungsteam zeitnah eine Nachbesprechung erfolgen.

### Empfehlung 5: psychosoziale Unterstützung anbieten

Eine psychosoziale Unterstützung rund um den Besuch von Kindern sollte gewährleistet sein, z. B. über Psycholog:innen, Seelsorger:innen oder Kriseninterventionsteams. Den Intensivstationen wird empfohlen, sich im Vorfeld mit Unterstützungsangeboten vertraut und ggf. mit den Institutionen bekannt zu machen. Es ist hilfreich, die Kontaktdaten (Flyer) dieser Einrichtungen den Mitarbeiter:innen, Angehörigen, Eltern zugänglich zu machen. Ebenso ist es wichtig abzuklären, wer die Kosten trägt.

### Empfehlung 6: in palliativen Situationen besonders begleiten

Im Fall einer palliativen Situation und bei bereits verstorbenen Patient:innen sollte mit Kindern das offene Gespräch über die Themen Sterben, Tod und Trauer gesucht werden. Hilfreich ist dabei ein Austausch auf Augenhöhe, bei dem Kinder ihre eigenen Vorstellungen und Phantasien einbringen können. Sofern vorhanden, sollten palliativmedizinische Kompetenzen einer Klinik eingebunden werden, z. B. Palliativstationen, Palliativdienste, Palliativvisiten oder qualifizierte Mitarbeiter:innen.

### Empfehlung 7: in Notfallsituationen eine kindgerechte Begleitung ermöglichen

Eine psychosoziale Betreuung von Kindern spielt auch in Notaufnahmen, Kindernotaufnahmen sowie in wenig planbaren Situationen eine große Rolle. Da hier meist wichtige Vorbereitungszeit fehlt, sollte ein Mitglied des Behandlungsteams und/oder eine psychosoziale Fachkraft einen sicheren Kinderbesuch ermöglichen, ad hoc vorbereiten, begleiten und einen Nachsorgekontakt vermitteln.

### Empfehlung 8: Führung – den richtigen Rahmen für Kinderbesuche schaffen

Der Besuch von Kindern auf einer Intensivstation ist auch eine Führungsaufgabe. Das Management sollte die Mitarbeiter:innen für die Wichtigkeit des Themas sensibilisieren und motivieren, gute Lösungen zu schaffen. Eine kontinuierliche Schulung des Teams und Möglichkeiten zur Reflexion spielen dabei eine wichtige Rolle. Auch sollten mögliche Unsicherheiten und Belastungen im Team Berücksichtigung finden.

### Empfehlung 9: Qualitäts- und Risikomanagement einbinden

Damit der Besuch von Kindern auf der Intensivstation für alle erfolgreich verläuft, braucht es ein proaktives Qualitäts- und Risikomanagement und eine strukturierte Qualitätssicherung. Mögliche Kontraindikationen sollten besprochen und abgewogen werden. Der Kinderbesuch sollte im Rahmen einer Standard Operating Procedure (SOP) definiert werden, um die gewünschte Qualität des Besuchs und den Prozess festzulegen.

### Empfehlung 10: den Kinderbesuch und Angehörigengespräche dokumentieren

Eine Anamnese der wichtigsten Bezugspersonen, die Gespräche zum Einbezug von Kindern sowie die Planung und Durchführung des Besuchs sollten lückenlos dokumentiert werden. Alle Mitarbeiter:innen sollten Zugriff auf diese Informationen haben.

## Fazit für die Praxis

- Kinder können Besuche in Bereichen der medizinischen Intensiv‑, Notfall- und Palliativversorgung verarbeiten, wenn sie diese wünschen und altersgerecht informiert und vorbereitet werden.
- Sie stellen kein erhöhtes Infektionsrisiko für die Patient:innen dar, wenn die geltenden Hygieneanforderungen eingehalten werden.
- Ob ein Besuch zweckdienlich, durchführbar und von Kind und Patient:in gewollt ist, sollte im Vorfeld in Gesprächen mit den Betroffenen und ihren Angehörigen geklärt werden.
- Das Management und die Mitarbeiter:innen in den genannten Versorgungsbereichen sollten einen als sinnvoll evaluierten Besuchswunsch unterstützen und ermöglichen. Weitere psychosoziale Berufsgruppen sollten hierbei ggf. einbezogen und die Übernahme der damit verbundenen Kosten geklärt werden.
- Zur Vorbereitung und Durchführung des Besuchs existieren 10 konkrete Empfehlungen und ein unterstützender Handlungsalgorithmus, die die Bedarfe aller Beteiligten adressieren und einen bestmöglichen Ablauf unterstützen.

## Footnotes

1 An dieser Stelle wird verblindet auf die Originalveröffentlichung hingewiesen.

## References

[1] Allen L, Jones C, Fox A, Copello A, Jones N, Meiser-Stedman R (2021). The correlation between social support and post-traumatic stress disorder in children and adolescents: a meta-analysis. J Affect Disord.

[2] Arbeitskreis „Krankenhaus- & Praxishygiene“ der AWMF (2019). Hygieneanforderungen in der Intensivmedizin. AWMF-Register Nr. 029/028, Klasse S1.

[3] Bensch S, Heyd C (2020). Kinder als Besucher auf Intensivstation. Empirische Analyse zur Implementation eines Konzepts im deutschen Krankenhaus. Krankenhaus.

[4] Berti D, Ferdinande P, Moons P (2007). Beliefs and attitudes of intensive care nurses toward visits and open visiting policy. Intensive Care Med.

[5] Brauchle M, Deffner M, Banaschak S, Brinkmann A, Brüning T, Dehner S, Dubb R, Filzhoefer D, Finkeldei S, Gatzweiler B, Gerlach A, Hermes C, Heyd C, Hoffmann M, Jeitziner MM, Johnen T, Kaltwasser A, Kern T, Knochel K, Krüger L, Melching M, Michels G, Müller-Wolff TT, Pelz S, Plener P, Pönicke C, Schäfer A, Rudolph J, Schindele D, Seidlein AH, Simon A, Ufelmann M, Nydahl P (2022) Empfehlungen „Kinder als Angehörige und Besuchende auf Intensivstationen, pädiatrischen Intensivstationen, IMC-Stationen und in Notaufnahmen“. https://www.divi.de/empfehlungen/publikationen/intensiv-und-notfallpflege. Zugegriffen: 16. Nov. 2022

[6] Brauchle M, Deffner M, Nydahl P, ICU Study Group (2023). 10 recommendations for child-friendly visiting policies in critical care. Intensive Care Med.

[7] Clarke C, Harrison D (2001). The needs of children visiting on adult intensive care units: a review of the literature and recommendations for practice. J Adv Nurs.

[8] Deutschsprachige Gesellschaft für Psychotraumatologie S2k-Leitlinie „Diagnostik und Behandlung von akuten Folgen psychischer Traumatisierung“. https://register.awmf.org/assets/guidelines/051-027l_S2k_Diagnostik_Behandlung_akute_Folgen_psychischer_Traumatisierung_2019-10.pdf. Zugegriffen: 16. Nov. 2022

[9] Fumagalli S, Boncinelli L, Nostro LA, Valoti P, Baldereschi G, Di Bari M, Ungar A, Baldasseroni S, Geppetti P, Masotti G, Pini R, Marchionni N (2006). Reduced cardiocirculatory complications with unrestrictive visiting policy in an intensive care unit: results from a pilot, randomized trial. Circulation.

[10] Knutsson S, Bergbom I (2016). Children’s thoughts and feelings related to visiting critically ill relatives in an adult ICU: A qualitative study. Intensive Crit Care Nurs.

[11] KRINKO (2016). Händehygiene in Einrichtungen des Gesundheitswesens. Bundesgesundheitsbl.

[12] KRINKO (2021). Anforderungen an die Infektionsprävention bei der medizinischen Versorgung von immunsupprimierten Patienten: Empfehlung der Kommission für Krankenhaushygiene und Infektionsprävention (KRINKO) beim Robert Koch-Institut. Bundesgesundheitsblatt Gesundheitsforschung Gesundheitsschutz.

[13] Krüger L, Mannebach T, Rahner M, Timpe F, Wefer F, Nydahl P (2022). Learning in one minute: survey of the one minute wonder network. Med Klin Intensivmed Notfmed.

[14] Krüger L, Mannebach T, Wefer F, Bolte C (2021). One minute wonder—Inservice training during working hours. HBScience.

[15] Lautrette A, Darmon M, Megarbane B, Joly LM, Chevret S, Adrie C, Barnoud D, Bleichner G, Bruel C, Choukroun G, Curtis JR, Fieux F, Galliot R, Garrouste-Orgeas M, Georges H, Goldgran-Toledano D, Jourdain M, Loubert G, Reignier J, Saidi F, Souweine B, Vincent F, Barnes NK, Pochard F, Schlemmer B, Azoulay E (2007). A communication strategy and brochure for relatives of patients dying in the ICU. N Engl J Med.

[16] Salmon K, Bryant RA (2002). Posttraumatic stress disorder in children. The influence of developmental factors. Clin Psychol Rev.

[17] Youngner SJ, Coulton C, Welton R, Juknialis B, Jackson DL (1984). ICU visiting policies. Crit Care Med.

[18] Rainer J (2012). Child visitor, adult ICU. Nurs Manage.

[19] RKI (2007). Empfehlung zur Prävention nosokomialer Infektionen bei neonatologischen Intensivpflegepatienten mit einem Geburtsgewicht unter 1500 g. Bundesgesundheitsbl.

